# Pilot randomized controlled trials in the orthopaedic surgery literature: a systematic review

**DOI:** 10.1186/s12891-018-2337-7

**Published:** 2018-11-24

**Authors:** Bijal Desai, Veeral Desai, Shivani Shah, Archita Srinath, Amr Saleh, Nicole Simunovic, Andrew Duong, Sheila Sprague, Mohit Bhandari

**Affiliations:** 1 Faculty of Health Sciences, McMaster University, Hamilton, ON Canada; 2 Schulich School of Medicine and Dentistry, Western University, London, ON Canada; 3 Faculty of Medicine, University of Toronto, Toronto, ON Canada; 4 Department of Health Research Methods, Evidence, and Impact (HEI), Division of Orthopaedic Surgery, McMaster University, Hamilton, ON Canada; 5 Department of Surgery, Division of Orthopaedic Surgery, McMaster University, Hamilton, ON Canada

**Keywords:** Orthopaedic, Surgery, RCT, Feasibility, Pilot study, Definitive trial

## Abstract

**Background:**

The primary objective of this systematic review is to examine the characteristics of pilot randomized controlled trials (RCTs) in the orthopaedic surgery literature, including the proportion framed as feasibility trials and those that lead to definitive RCTs. This review aim to answer the question of whether pilot RCTs lead to definitive RCTs, whilst investigating the quality, feasibility and overall publication trends of orthopaedic pilot trials.

**Methods:**

Pilot RCTs in the orthopaedic literature were identified from three electronic databases (EMBASE, MEDLINE, and Pubmed) searched from database inception to January 2018. Search criteria included the evaluation of at least one orthopaedic surgical intervention, research on humans, and publication in English. Two reviewers independently screened the pool of pilot trials, and conducted a search for corresponding definitive trials. Screened pilot RCTs were assessed for feasibility outcomes related to efficiency, cost, and/or timeliness of a large-scale clinical trial involving a surgical intervention. The quality of the pilot and definitive trials were assessed using the Checklist to Evaluate a Report of a Non-Pharmacological Trial (CLEAR NPT).

**Results:**

The initial search for pilot RCTs yielded 3857 titles, of which 49 articles were relevant for this review. 73.5% (36/49) of the orthopaedic pilot RCTs were framed as feasibility trials. Of these, 5 corresponding definitive trials (10.2%) were found, of which four were published and one ongoing. Based on author responses, the lack of a definitive RCT following the pilot trial was attributed to a lack of funding, inadequacies in recruitment, and belief that the pilot RCT sufficiently answered the research question.

**Conclusions:**

Based on this systematic review, most pilot RCTs were characterized as feasibility trials. However, the majority of published pilot RCTs did not lead to definitive trials. This discrepancy was mainly attributed to poor feasibility (e.g. poor recruitment) and lack of funding for an orthopaedic surgical definitive trial. In recent years this discrepancy may be due to researchers saving on time and cost by rolling their pilot patients into the definitive RCT rather than publish a separate pilot trial.

**Electronic supplementary material:**

The online version of this article (10.1186/s12891-018-2337-7) contains supplementary material, which is available to authorized users.

## Background

Definitive randomized controlled trials (RCTs) exist to demonstrate unmistakable evidence of a certain inventions benefit on a patient [1]. Although they are very impactful for clinical practice are typically expensive and time-consuming [2]. Given the resources and time, investigators often conduct pilot trials designed with an aim to demonstrate the feasibility of the larger-scale definitive trial [3]. Pilot trials can identify possible challenges, predict costs, and fine-tune study design. In addition, by demonstrating feasibility, a successful pilot trial can be used to leverage momentum and definitive trial funding [2].

Effective pilot trials have a well-defined set of objectives to assess feasibility.^3^ Feasibility is assessed in terms of whether the intervention of interest, trial design, and protocol can be successfully implemented and completed by the researchers [3]. Feasibility can be determined at the program level, study level, and site or investigator level. Program level feasibilities include determining the prevalence of particular diseases in a particular region and include clinical and epidemiological trials [4]. Study level feasibilities are centered on assessing whether a specific clinical trial can be conducted in a country or region [3]. Site or investigator level feasibility trials focus on identifying challenges and probable solutions with respect to the investigator and clinical aspects of the trial (drug dosages, actual study population, recruitment and follow-up, usage of assessment tools, etc.) [4].

Despite the benefits of pilot trials, previous literature has demonstrated that they do not always lead to a definitive trial. In 2004, Lancaster et al reviewed four general medicine journals and three specialist journals and identified 90 pilot studies published from 2000 to 2001; of which 45 reported the intention to carry out further work [1]. However, in 2010, Arain et al. found that only eight out of the 45 were followed by a larger, definitive study [5]. The impact of pilot data and subsequent research remains to be evaluated in the orthopaedic surgical literature.

This systematic review assessed the quality of pilot RCTs and frequency of ensuing definitive RCTs in the orthopaedic surgical literature. The primary objectives of this review were to: 1) assess feasibility outcomes across pilot trials in the orthopaedic surgery literature; 2) identify the proportion of pilot trials that lead to and how they inform definitive RCTs, and 3) evaluate the quality and frequency of pilot trials over time.

## Methods

### Identification of RCTs

EMBASE, MEDLINE and Pubmed were searched for relevant articles published from database inception until January 25, 2018 (Additional file 1). All search results were imported into the Mendeley Reference Manager software (Elsevier Publishing, 2013) to remove all duplicate trials.

Once the final pool of included pilot RCTs was determined, an additional search was conducted in the same electronic databases in an attempt to find corresponding definitive trials. If a literature search of titles was unsuccessful, other trials conducted by at least one of the authors after the pilot were considered. The secondary search was conducted using key terms used in the pilot RCT. Additionally, clinicaltrials.gov, an online database of ongoing clinical trials, was reviewed to determine if the previously identified pilot RCTs had a definitive trial in progress. Finally, if no definitive trial was found using these methods, the pilot RCT authors were contacted by email and asked whether a definitive trials was ongoing, published, or submitted for publication.

### Eligibility criteria

Trails had to be defined explicitly and reported as pilot trials within the paper itself to be included in this review. Trials reported as pilot RCTs were deemed eligible for this review if they: 1) included an orthopaedic surgical intervention, 2) included a drug that was used intra-operatively at the site of surgery/fracture, or 3) evaluated the difference between two surgical interventions or surgical vs. non-surgical orthopaedic interventions. Only clinical trials in humans published in English were included. RCTs were excluded if they were: 1) non-pilot RCT designs (including small trials not reported as pilot trials) 2) trial interventions were exclusively non-surgical including physiotherapy, exercise regimens, post-operative rehabilitation, anesthesia, post-operative pain management interventions, or 3) trial interventions were surgical procedures not related to orthopaedics (e.g. oral, urology, and ocular surgeries), and 4) drugs and supplements administered orally (intravenously administered during surgery were included).

### Screening

**Fig. 1:**
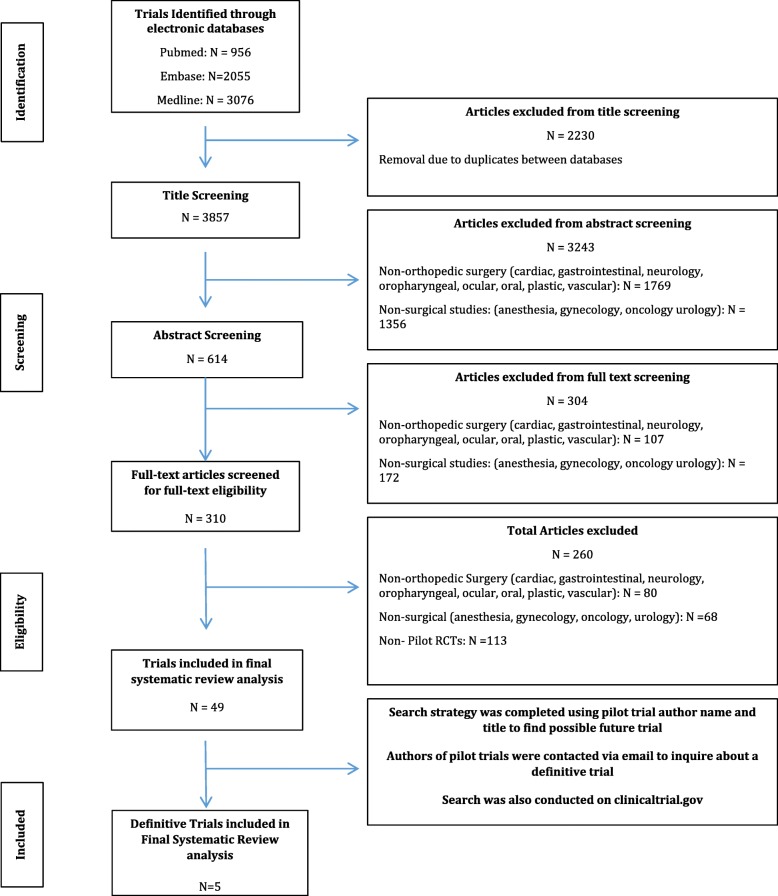
Flow diagram of the search and screening strategies to define the final pool of trails

Articles were independently screened in duplicate at the title, abstract, and full-text stage by decisions were independently recorded in a spreadsheet (Microsoft Excel, 2015). In order to ensure comprehensive screening, an article was progressed to the next screening stage if at least one reviewer had noted that the article should be included, and illustrated as a flow diagram and checklist in Fig. 1 below. All disagreements were resolved by consensus during the full-text screening phase in consultation with a third senior reviewer (AD).

### Data abstraction

Pilot and definitive trial data, such as the country, number of patients in the RCT, orthopaedic condition being treated, orthopaedic intervention(s), controls, primary and secondary outcomes, percentage of patients that were lost to follow-up, follow-up schedule, and feasibility objectives were abstracted. In addition, for the definitive trials, any changes in interventions, controls, primary and secondary outcomes, or patient sample from the pilot trial were noted. For definitive RCTs, the time elapsed between the date of publication of the pilot and definitive trial and whether or not the sample size was calculated based on event rates from the pilot trial were determined.

### Assessment of feasibility

Feasibility trials were defined as trials with a primary purpose of piloting the protocol to inform a definitive trial. In order to distinguish between pilot trials created solely for investigating the efficacy of interventions compared to feasibility trials, specific reference to feasibility objectives were evaluated. Feasibility objectives include determining the preliminary efficacy of a surgical intervention as well as the safety of the intervention, accurate event rates for a definitive sample size, the cost of a large scale clinical trial, patient recruitment rates, trial design, randomization procedure, and ability to maintain blinding.

### Assessment of methodological quality

The reviewers (BD, VD, ALS, and SS) independently assessed the quality of each included pilot and definitive trial in duplicate using the Checklist to Evaluate A Report of Non-Pharmacological Trial (CLEAR NPT). The CLEARN NPT is designed for the critical appraisal of RCTs in nonpharmacological and surgical trials [6]. The original checklist was modified, where the question regarding patient adherence was omitted as all our trials evaluated a single, one-time surgical intervention. As the original checklist did not provide a scoring method, the criteria employed by Somford et al. was adopted to provide a modified CLEAR NPT (Additional file 2) [6]. The maximum CLEAR NPT score was 18, whereby a score of 0–6 indicated a low quality trial, 7–12 indicated a medium quality trial, and 13–18 indicated a high quality trial.

### Statistical analysis

A kappa (κ) statistic was used to determine agreement at all stages of article screening with 95% confidence intervals (CI) [7]. An intraclass correlation coefficient (ICC) was calculated for the purpose of evaluating inter-rater reliability for the CLEAR NPT quality assessment. Agreement for both the κ and ICC was categorized as follows: > 0.90 indicated an almost perfect level of agreement, 0.80 < 0.90 strong agreement, 0.60 < 0.79 moderate agreement, 0.40 < 0.59 weak agreement, 0.21 < 0.39 minimal agreement, and 0.0 < 0.20 no agreement [8].

A t-test was performed using an online statistical calculator (Vassal Stats) to compare trial quality between pilot and definitive trials and a Pearson’s r correlation was calculated to determine if there was a relationship between number of studies and quality of pilot RCTs over time. A *p*-value less than 0.05 was considered significant. Descriptive statistics including means, proportions, standard deviations, and CIs are reported. A meta-analysis was not performed given the broad heterogeneity of the trial designs, interventions, and outcome measures.

## Results

### Screening

The initial screening of online databases yielded 3857 articles after the removal of 2230 duplicates. After title, abstract, and full text screening, 49 pilot RCTs were included (Fig. 1 and Additional file 3). Of these, we identified five definitive trials (one of which is still ongoing) that corresponded to the original published pilot trial. Inter-reviewer agreement was high at all stages of screening (title, κ = 0.886 (95% CI 0.878 to 0.893); abstract, κ = 0.740 (95% CI 0.693 to 0.780); and full text, κ = 0.792 (95% CI 0.737 to 0.835)).

### Pilot trial characteristics

**Fig. 2:**
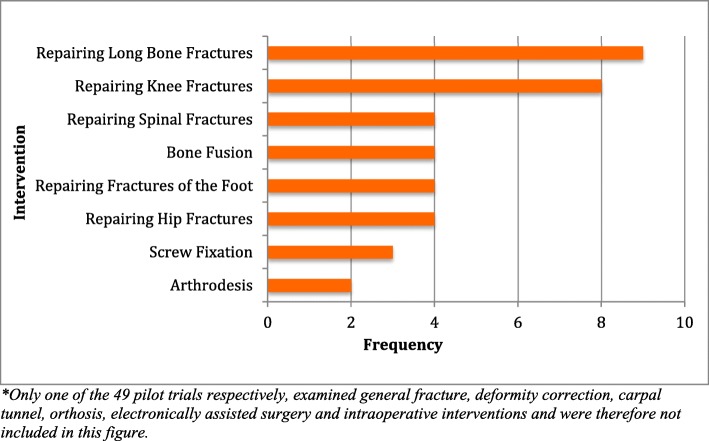
Frequency of various types of pilot RCT interventions in each intervention category

Pilot trials were commonly published from the UK and Canada (22 and 16%, respectively) (Tables 1 and 2). A total of 2117 patients were recruited across all pilot RCTs, and 5.84 ± 10.9% of patients, on average, were lost to follow up. The greatest proportion of pilot trials (59.2%, 29/49) focused on surgical fracture repair, including long bone, knee, spinal, foot and hip fractures (Fig. 2). As classified by the World Bank, 40 of the pilot trails were conducted in high-income countries, 6 were classified as middle income and 3 were classified as low income [9].

**Table 1:** Characteristics of the Included Pilot RCTs

Title	Country	# of patients	Condition	Type of Surgery	Control	Patient-Reported Outcomes	Clinician Reported Outcomes	Primary Outcome	Secondary Outcome	Loss to follow-up (%)	Length of follow-up	Feasibility Category	Framed as feasibility?	Definitive Trial Published?	CLEAR-NPT Score
Hamdy, R. C et al., (2009) [15]	Canada	52	Lower extremity limb lengthening and deformity correction.	Ilizarov technique	Sterile saline solution	Faces Pain Scale-revised, Adolescent Pediatric Pain Tool, Pediatric Quality of Life Inventory, Gillette Functional Assessment Questionnaire	n/a	Pain, quality of life, functional mobility	Adverse effects	3%	3 mo	Study design	Yes	Yes	17
Vaccaro, A. R et al., (2005) [16]	USA	12	Iliac crest autograft in posterolateral lumbar fusions	Op-1 putty (rhbmp-7) as an adjunct	No control group	Oswestry scale and SF-36	Radiographic	Fusion success rate	Cost effectiveness and pain	0%	24 mo	Safety & efficacy	Yes	No	11
Ekrol, I et al., (2008) [17]	UK	30	Distal radial fractures.	RhBMP-7 as an alternative to bone graft healing	No control group	n/a	Radiographic, clinical and functional outcomes	Functional outcome	Complications	0%	12 mo	Efficacy	Yes	No	18
Lerner, T et al., (2009) [18]	Germany	40	Adolescent idiopathic scoliosis.	Graft extenders in scoliosis surgery	No Control Group	VAS pain score	Biopsies	Fusion success rate	Pain	0%	48 mo	Efficacy	Yes	No	15.5
Marks, P et al., (2008) [19]	Canada	40	ACL reconstruction	Comparing the Mitek bone–tendon– bone cross pin and bioabsorbable screw	No control - comparative trial	International Knee Documentation Committee (IKDC) Knee Ligament Standard Evaluation, Mohtadi’s ACL Deficiency Quality of Life	Lateral x-Ray	Clinical outcomes	Length of surgery	20%	24 mo	Efficacy	Yes	No	16.5
Shamji, M. F et al., (2014) [20]	Canada	23	Thoracolumbar burst	Applying a brace to treat thoracolumbar bursts	No brace	VAS pain score	Radiographic, clinical & functional outcome	Average length of hospital stay & cost	Pain & adverse effects	0%	6 mo	Efficacy	Yes	No	14.5
Carson, J. L et al., (1998) [21]	UK	84	Hip fracture	Blood transfusion during hip fracture	No Control- comparative trial	Phone interviews of family or patient	Various methods to evaluate adverse effects (CT scan, ultrasound, autopsy)	Mortality & ability to walk 10 ft across a room	Functional status, residence status, in-hospital myocardial infarction,thromboembolism, stroke and pneumonia.	3%	60 days	Patient recruitment	Yes	No	16.5
Vaccaro, A. R et al., (2003) [22]	USA	12	Iliac crest autograft in posterolateral lumbar fusions	Op-1 putty (rhbmp-7) as an adjunct	No control group	n/a	Radiographic & clinical	Fusion success rate	Adverse effects	0%	24 mo	Safety & efficacy	Yes	No	13.5
Vaccaro, A. R et al., (2004) [23]	USA	36	Symptomatic degenerative spondylolisthesis	Spinal stenosis	Autograft	n/a	Radiographic & clinically	Fusion success rate	Well-being/Quality of life	5%	1 yr	Safety	Yes	No	18
Glazebrook, M et al., (2013) [24]	Canada	24	Ankle and Hindfoot Arthrodesis	B2A-Coated Ceramic Granules (Amplex) Compared to Autograft	Autograft	Computerized tomography & Ankle Osteoarthritis Scale scores	n/a	Bone healing at site of fusion	n/a	0%	12 mo	Safety & efficacy	Yes	No	17
Mauffrey, C et al., (2012) [25]	UK	24	Tibial fracture	Arthrodesis surgery	No control - comparative trial	Functional outcome questionnaire (DRI)	n/a	DRI	Olerud and Molander Ankle Score (OMAS)10 and the EuroQol EQ-5D generalized health outcome questionnaire	0%	12 mo	Efficacy	Yes	No	18
Darmanis, S et al., (2007) [26]	UK	40	Knee arthroplasty	Knee arthroplasty	No laser	Fisher’s Exact test and the Mann-Whitney U test	n/a	Fisher’s Exact test and the Mann-Whitney U test	n/a	0%	n/a	Efficacy	Yes	No	15.5
Buse, G. L et al., (2014) [27]	Canada	60	Hip fracture	Accelerated surgery	No	Functional Independence Measure & SF-36	Troponin measurement & confusion Assessment Method.	Proportion of eligible patients randomly assigned, completeness of follow-up & timelines of accelerated surgery	Major perioperative complication	0%	18 mo	Sample size	Yes	No- Ongoing Definitive Trial	15
Adolfsson, L et al., (2001) [28]	Sweden	53	Waist	Percutaneous Acutrak screw fixation.	Immobilization in a below elbow plaster cast for 10 weeks	n/a	Radiographic	Assessment of union	Failure	1%	10 wks	Efficacy	Yes	No	16.5
Kang, P et al., (2012) [29]	China	77	Femoral	Multiple drilling core decompression combined with systemic alendronate as a femoral head-preserving procedure	Multiple drilling core decompression	THA & Harris score	Radiographic	Efficacy of combined treatment	Reduction in disease progression	10%	48 mo	Safety & efficacy	Yes	No	17
Moseley, J. B et al., (1996) [30]	USA	10	knee	Arthroscapic surgery of the knee	Placebo (sterile saline)	Arthritis Impact Measurement Scale 34 and the SF-36	n/a	Evaluate the placebo effect as part of a pilot trial preceding a randomized, controlled trial of arthroscopic treatment of osteoarthritis	Ability to find and recruit eligible subjects; developing and testing measurement instruments; ability to retain patients for at least 6 months; determine feasibility of completing trial	0%	12 mo	Ability of patients to complete the trial; ability to maintain blindness	Yes	No	18
Chhabra, H. S et al., (2009) [31]	India	5	Spinal cord	Olfactory mucosal transplantation into the spinal cord	No	American Spinal Injury Association (ASIA) Impairment Scale (AIS), Spinal Cord Independence Measure, Beck Depression Inventory scores and life impact scores on International Spinal Cord Injury Scale	MRI	Autologous olfactory mucosal transplantation therapy	n/a	0%	18 mo	Safety & efficacy	Yes	No	16
Wang, Y et al., (2015) [32]	China	21	Foot	Total-contact casting (TCC)	No	Quantitive sensory Testing (temperature, pain, light touch, perception)	Radiographic	Comparing the effectiveness of arthrodesis plus TCC with TCC alone for the prevention, treatment and recurrence of midfoot ulcerations	Sample size	0%	12 mo	n/a	No	No	18
Zou, J et al., (2013) [33]	China	94	Tibial	Minimally invasivepercutaneous plate osteosynthesis	Comparative trial, control group was treated with ORIF	n/a	Radiographic	Functional status	Sample size	0%	12 mo	n/a	No	No	16.5
Zehir, S et al., (2015) [34]	Turkey	45	Clavicular	Minimally invasive plating fixation	Mini invasive plating	(DASH) Quick Disability of the Arm, Shoulder and Hand	Radiographic	Efficacy	Sample size	0%	12 mo	Efficacy	Yes	No	16.5
Pang, H. N et al., (2011) [35]	Singapore	140	Knee	Knee arthroplasty	Resection technique	Knee Society Score [20], Oxford Knee Questionnaire [36] and SF-36 Questionnaire	Radiographic	Efficacy	Unclear	0%	24 mo	Efficacy	Yes	No	18
Wondrasch, B et al., (2009) [36]	Austria	21	Femoral	Matrix associated autologous chondrocyte implantation	Delayed weightbearing	Knee Documentation Committee (IKDC), the Tegner activity scale, and the Knee Injury and Osteoarthritis Outcome Score (KOOS)	Radiological outcome was evaluated by the MOCART score and the size and amount of bone marrow edema and effusion	Efficacy	n/a	0%	24 mo	Efficacy	Yes	No	14
De Sèze, M. P et al., (2011) [11]	France	28	ankle	ankle foot orthosis	Standard ankle-foot orthosis	Pain evaluation on an analogical visual scale	n/a	Gain ratio at day 30		0%		Efficacy	Yes	No	16
Kraus, V. B et al., (2012) [37]	USA	11	Knee	ACL	Placebo (sterile saline)	Standardized Knee Injury and Osteoarthritis Outcome Score (KOOS) questionnaire	n/a	Efficacy	Sample size	0%	1 mo	Efficacy	Yes	No	16
Flow Investigators (2011) [38]	Canada	111	General open fractures	Fracture healing	Saline	SF-12 and EQ-5D, respectively (12-item questionnaire that measures health-related quality of life in 8 domains)	n/a	Reoperation rates	Infection & wound healing problems	11%	12 mo	Efficacy & large scale trial	Yes	Yes	18
Lindsey, R. W et al., (2006) [39]	USA	10	General long bone fractures	Grafting long bone fractures	No	n/a	Radiographic	Effectiveness of a composite graft consisting of demineralized bone matrix (DBM) putty enriched with aspirated bone marrow	Sample size	0%	24 mo	Efficacy	Yes	No	16
Costa, M. L et al., (2003) [40]	UK	28	Achilles tendon	Achilles tendon ruptures	Serial plaster casting	n/a	Ultrasound	Safety	Sample size	29%	12 mo	Safety	Yes	Yes	18
Mahowald, M. L et al., (2009) [41]	USA	11	Advanced rheumatoid arthritis and osteoarthritis	IA-BONT/A	Saline Placebo	Patient global assessment of change was measured with a validated 7-point verbal descriptor scale & WOMAC	n/a	Efficacy and safety of IA-BoNT/	Decreases in pain and functional improvement	13%	1 mo	Safety & efficacy	Yes	No	12
Paterson, K et al., (2013) [42]	Australia	37	Knee	PA-PRP	Hyaluronic acid	VAS pain score	n/a	Recruitment and safety data	Symptomatic and functional changes following treatment	17%	4 and 12 wks	Efficacy	Yes	No	18
Zehir, S et al., (2014) [43]	Turkey	64	Distal radius fractures	Sonoma Wrx	Standard volar locking plate fixation	n/a	Radiographic	Reliability and efficacy	Complication prevention	0%	12–13 mo	Efficacy	Yes	No	13.5
Hey, H et al., (2013) [44]	Singapore	7	Spinal	Hybrid surgery	Anterior cervical discectomy and fusion + artificial disc replacement	Complications and functional scores (VAS, NDI, EQ-5D health score and index)	n/a	Clinical differences between three groups	n/a	0%	2 wks	Safety & efficacy	Yes	No	10.5
Kuo, L. C et al., (2013) [45]	Taiwan	22	Radial	Early digit mobilization	Home programmes	Manual Ability Measure-36 (MAM-36) to assess their self-awareness of manual abilities	Radiographic	Differences in functional outcome	n/a	0%	12 wks	Efficacy & cost	Yes	No	18
Abbott, A et al., (2013) [46]	Sweden	17	Cervical	With post-operative cervical collar usage	Without post-operative cervical collar usage	Falls Efficacy Scale (patients measuring completing tasks without falling)	Radiographic	Sample size feasibility	Physical, functional, and quality of life-related outcomes	45%	6 wks	Cost & sample size	Yes	No	12
McMorland, G et al., (2010) [47]	Canada	120	SCIATICA	Surgical microdiskectomy	Standardized chiropractic spinal manipulation	McGill Pain Questionnaire, Aberdeen Back Pain Scale, and Roland-Morris Disability Index)	n/a	Recruitment and randomization process, choice of outcome measures, and effect size for definitive trial	Compare the clinical efficacy	0%	1 yr	Sample size patient recruitment randomization	Yes	No	18
Ringel, F et al., (2012) [48]	Germany	60	Spinal	Robot-assisted (RO) implantation	Freehand (FH) conventional technique	n/a	CT scan	Evaluate accuracy of techniques	n/a	0%	No follow up	Efficacy	Yes	No	16
Boonen, S et al., (2002) [49]	Belgium	33	Proximal Femoral Fracture	Administration of rhIGF-I/IGFBP-3	Placebo	Kerr-Atkins score for pain and function	Radiographic	Pain and function	Musculoskeletal effects	0%	6 mo	Safety	Yes	No	17
Griffin, D et al., (2014) [50]	UK	151	Calcaneal fractures	Surgery by open reduction and internal fixation	Non-operative treatment	Kerr-Atkins calcaneal fracture score	Radiographic	Pain and function	Complications	5%	24 mo		No	No	18
Guo, Q et al., (2013) [51]	China	90	Intertrochanteric fractures	Percutaneous compression plate	Proximal femoral nail anti-rotation	n/a	Radiographic	Clinical effectiveness	Complications	0%	2 yrs		No	No	16.5
Storey, P et al., (2013) [52]	UK	49	Carpal Tunnel	C-Trac splints	Conventional resting splint	n/a	Radiographic	Levine questionnaire scores	Grip, pinch and sensation scores	0%	1 yr		No	No	14
Zhang, Y. Z et al., (2011) [53]	China	22	Abnormalities with hip joint	Navigation template implantation	Conventional THA	n/a	CT scan	Unclear	Unclear	0%	12 to 18 mo		No	No	13
Nejrup, K et al., (2008) [54]	Denmark	43	Knee osteoarthritis	Gold implantation	Sham implantation	WOMAC, Knee Society Clinical Rating System	Radiographic	Knee pain, stiffness and function	Time from implant until effect and migration potential of implanted gold beads.	7%	0, 6 and 12 mo		No	No	18
Eskander, M. B. F et al., (1997) [55]	UK	44	Femoral neck fractures	Enoxaparin	Application of intermittent calf compression garments	n/a	CT scan	Unclear	Unclear	0%	6 wks		No	No	16.5
Jordan, R et al., (2014) [56]	UK	24	Tibial plateau fractures	Balloon osteoplasty	Traditional methods	Oxford Knee score and SF12 questionnaire at 3, 6 and 12 month	CT scan	Quality of reduction based on the post-operative CT scan	Surgical complication & patient satisfaction	Not stated	3, 6 and 12 mo		No	No	18
Kearney, R. S et al., (2011) [57]	UK	52	Achilles tendon rupture	Surgical repair	Non-surgical repair	Disability rating index EQ-5D, Achilles Total Rupture Score	n/a	Estimate of effect size for the functional outcome	Assess the use of a comprehensive cohort research design	10%	2 wks, 6 wks, 3 mo, 6 mo & 9 mo	Study design	Yes	No	10
Sabeti, M et al., (2014) [58]	Austria	20	Calcific rotator cuff tendinitis	Intraoperative ultrasound	Needling and palpating techniques	n/a	Radiographic	Notable potential clinical differences between the two groups	Clinical improvement using ultrasound	10%	2 and 6 wks and the 9 mo		No	No	13
Dutton, T et al., (2012) [59]	UK	48	Knee fractures	Retransfusion drain	No drain	n/a	Measure of transfusion rate	Which patients benefit most from drains	n/a	0%	No follow up		No	No	12.5
Sardar, Z et al., (2015) [60]	Canada	24	Spinal fracture	B2A peptide–enhanced ceramic granules (Prefix)	Autogenous iliac crest bone graft (ICBG)	n/a	Radiographic	Safety and efficacy	Fusion rate	0%	6 wks, 3, 6, and 12 mo after surgery.		No	No	14.5
Capa-Grasa, A et al., (2014) [61]	Spain	40	Carpal Tunnel	Ultra-MIS	Mini-open Carpal Tunnel Release	Quick-DASH questionnaire	n/a	Safety and efficacy	Recruitment rates, compliance, completion, treatment blinding, personnel resources and sample size calculation	0%	3 mo	Safety, sample size & cost	Yes	Yes	17
Okcu, G et al., (2013) [62]	Turkey	40	Reverse obliquity fractures of the proximal femur	Long nail implant	Standard nail implant	n/a	Radiographic	Reoperation (fixation failure), 1 year mortality rate, function and mobility & union rate	Implant success	18%	12 to 20 mo		No	No	16

**Table 2:** Characteristics of Included Definitive trials

Pilot RCT Author	Definitive RCT Author	Country of Publication	Number. Of Patients	Change in Condition/Intervention	Primary Outcomes	Secondary Outcomes	Loss to Follow up (%)	Were Pilot Patients rolled into the definitive sample size?	Was the sample size calculation based on data from the pilot?	Number of months between publication of pilot trial and definitive trial	CLEAR NPT score:
Buse G. et al. (2014) [27]	Manach, Y. L. et al.,(ongoing) [63]	Canada	1200 (still recruiting)	No change	Major perioperative complication	Mortality, myocardial infarction, cardiac revascularization procedure	0.00	Unclear	Yes	68 (projeted end date July 2018)	18
Hamdy, R. C. et al. (2009) [15]	Hamdy, R. C. et al. (2016) [64]	Canada	125	No change	Quality of life (questionaires, filled out by parents and children) 4 PedsQL scales.	n/a	4.00	Unclear	No	78	18
Flow Investigators (2011) [38]	Bhandari M. et al. (2015) [65]	Canada	2551	No change	Reoperation, to treat an infection at the operative site or contiguous to it, manage a wound-healing problem or promote bone healing.	n/a	0.00	No	No	51	18
Costa, M. et al. (2003) [40]	Costa, M. et al. (2006) [66]	UK	48	No change	Time taken to return to normal activities, as reported by the patient	Complication rate in the treatment group	10.40	No	Yes	26	18
Capa-Grasa. et al. (2014) [61]	Rojo-Manaute, J. et al. (2016) [67]	Spain	92	No change	To compare the clinical outcomes of two surgical outcomes with primary carpal tunnel syndrome.	n/a	0.00	No	Yes	4	18

Primary and secondary outcomes of the pilot trials were divided into physician-reported and patient-reported outcomes. 65.3% (32/49) of all pilot RCTs used radiographic analysis, such as x-rays, MRIs, ultrasounds and CT scans. Patient-reported outcomes were recorded through self-reporting or interview style questionnaires. Questionnaires addressed outcomes such as quality of life, pain, function/independence, and emotional health. Of the pilot trials, 67.3% (33/49) made use of patient-reported questionnaires as tools for monitoring trial outcomes.

**Fig. 3:**
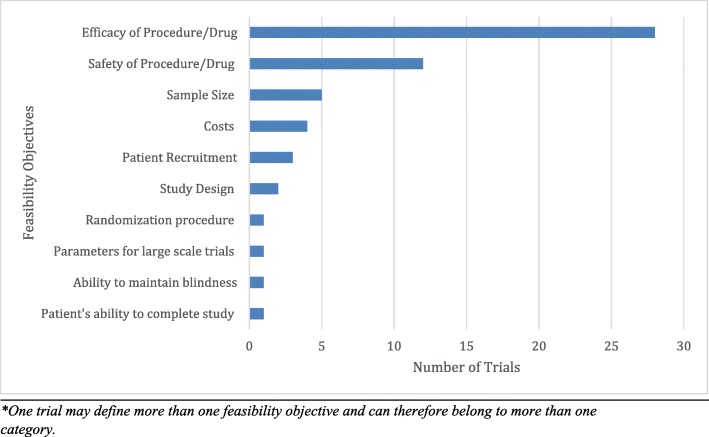
Number of RCTs that define each of these feasibility objectives in their pilot RCT

Overall, 73.5% (36/49) of the pilot RCTs found in the orthopaedic surgery literature were framed as feasibility trials (Table 1). The two most commonly explored feasibility objectives were safety and efficacy of an orthopaedic surgical intervention (Fig. 3). 26.5% (13/49) of pilot RCTs explored more than one feasibility objective. The pilot trials CLEAR NPT rating varied from 10 to 18. Only 3 of the 5 definitive RCTs included in this review determined their sample size based on their corresponding pilot trial. None of the definitive RCTs enrolled the pilot patients into the definitive trial. Additionally, 22.4% (11/49) of the pilot trials listed the efficacy/effectiveness of the surgical intervention as a primary outcome. Of these, only one led to a definitive trial.

### Definitive trial characteristics

Of the 49 identified pilot RCTs, five (10.2%) corresponding definitive RCTs were found (Table 2). On average, definitive trials were published at a mean of 4.25 years (3–7 years) after the pilot trial. The sample size of the pilot trial was 7.2% of the definitive trials. The total number of patients recruited to definitive RCTs was 4016, with one trial still recruiting participants (Table 2). Only one of these definitive trials was ongoing according to clinicaltrials.gov (31). Authors from 17 pilot trials (34.7%) responded to our email confirming that a definitive trial had not been published. Of these, 8 authors cited the following reasons for not conducting a definitive RCT: a lack of funding (12.5%), inability to meet recruitment targets (12.5%), preliminary efficacy of the intervention was not demonstrated (25.0%), the pilot study was thought to yield reliable results therefore eliminating the need for further investigation (50.0%).

### Trial quality

There was no correlation (*r* = − 0.1508, *p* = 0.5655) between number of studies and quality of pilot RCTs over time (Table 3). The overall quality of the pilot RCTs was relatively high (mean CLEAR NPT score 15.9 ± 1.53). Based on the CLEAR NPT scale, the highest quality pilot RCTs involved the treatment of arthrodesis and repair of knee fractures. All of the definitive RCTs were given a score of 18 and were therefore 2.6 points higher on the CLEAR NPT scale than their corresponding pilot trials (*p* < 0.01). The agreement among reviewers for the quality assessment was very high (ICC = 0.969 (95% CI 0.948 to 0.982)).

**Table 3:** Number and average quality of pilot RCTs over time of publication

Year	Number of Trials	Mean Clear NPT Score
1996	1	18.0
1997	1	16.5
1998	1	16.5
2001	1	16.5
2002	1	17.0
2003	3	16.5
2005	1	11.0
2006	1	16.0
2007	1	15.5
2008	3	17.5
2009	5	14.9
2010	2	17.0
2011	5	15.0
2012	4	15.9
2013	10	15.3
2014	6	15.7
2015	3	16.3
2016	0	n/a
2017	0	n/a
2018	0	n/a

## Discussion

Results from this systematic review demonstrate that the majority of orthopaedic surgical pilot RCTs were framed as feasibility trials, and that the pilot trials mostly evaluated site or investigator level feasibility. As expected, the quality of the corresponding definitive RCTs was higher than their respective pilot trial. Despite the majority (87%) of pilot RCTs being conducted in the high-income countries, the majority of the included pilot trials however, did not lead to a definitive RCT. In these cases, reasons cited included: a lack of funding, inadequate sample sizes, and that research questions were sufficiently answered in the pilot phase.

Similar to other fields of medicine, the majority of orthopaedic surgical pilot trials were not followed by a definitive trial. Arain et al. reviewed seven medical journals, including four general medicine journals (British Medical Journal, Lancet, the New England Journal of Medicine and the Journal of American Medical Association) and three specialist journals (British Journal of Surgery, British Journal of Cancer, British Journal of Obstetrics and Gynecology) to identify 54 pilot studies [5]. The authors reported a very low number of follow up studies, wherein only 14.8% (8/54) pilot studies yielded published definitive studies. Additionally, a systematic review published in 2017 by Kaur et al., looked at the quality of pilot studies within the Clinical Rehabilitation journal over the past 30 years, and they concluded that only 12% of their pilot studies led to a definitive trial [10].

The limited number of published pilot trials and corresponding definitive trials may be attributed to numerous factors. Firstly, the pilot may have demonstrated that a definitive trial was not feasible based on criteria established a priori (e.g. ability to recruit patients). However, we would expect that in some of these cases, researchers would amend their trial design, interventions, and outcomes to ensure feasibility in the definitive trial. Secondly, if found to be feasible, investigators may refrain from publishing their pilot trial and instead, roll the pilot patients into the definitive RCT to help save on time and costs. Trial methods papers and online registries are often used to first describe these trials. Thirdly, based on author responses in this review, definitive trials may not be feasible due to a lack of funding. In one case, the authors noted that their research question was answered by the pilot trial [11]. However, the published pilot did not provide a sample size calculation, and therefore, we cannot determine if the statistical power threshold was met for the primary outcome [12].

The majority of the orthopaedic surgical pilot trials found in this review posited feasibility objectives and were of relatively high quality. The first published pilot surgical trial was found in 1996, and since then, there has been an increase in the number of pilot RCTs published over time, with a relatively constant quality of trials up until 2013, with a decline in publications up until 2016. From 2016 to the end of our search in 2018, there were no orthopaedic surgical pilot RCTs published. This may be due to a more recent trend of trialists to roll their pilot patients into a definitive trial to save on costs and maximize recruitment. There may also be a lag in pilot publications in the past 3 years.

### Strengths and limitations

Strengths of this review include a broad systematic search and high agreement at all stages of screening and quality assessment. The main limitation is the minimal data available regarding the reasons why pilot trials have not led to definitive RCTs. There was a lack of response from authors, limiting further insight into barriers to definitive trials. Within the past 5 years, 13 of the 49 pilot RCTs and 4 of the 5 definitive trials were published. Thus, the inclusion of more recent pilot RCTs may be a limitation, as their current definitive trials may be underway, and/or not yet published. This potential source of bias was mitigated by searching the clinical trial registry, clinicaltrials.gov, for any records of ongoing definitive RCTs.

This review includes the use of the CLEAR NPT checklist to evaluate each pilot trial. Specifically within orthopedic literature, the quality of reporting RCTs using the CLEAR NPT is suboptimal, and that there is a need for improved surgical reporting [13]. However, in comparison to the CONSORT statement, the CLEAR NPT scale proves to be more useful in its analysis in interventions that require technical skill, with unique considerations in both conducting and reporting trials [14]. In this review, to account for methodological considerations, a modified CLEAR NPT scale was used instead to increase reliability and remove the necessity of including the Cochrane Risk of Bias Tool. The CLEAR NPT scale was modified, tested and optimized for orthopaedic trials, which was the focus of this paper.

## Conclusion

While the majority of pilot RCTs found in the surgical orthopaedic literature are framed as feasibility trials, most did not lead to definitive trials. The reported reasons include: minimal funding, the inability to recruit an adequate sample size and that the research questions were sufficiently answered in the pilot phase. Although, most pilot RCTs did not result in a definitive trial, this does not diminish the value of the pilot trial in determining feasibility.

## Additional files

Additional file 1: Search Strategy. (DOCX 63 kb)

Additional file 2: Modified Scoring of the CLEAR NPT Scale. (DOCX 87 kb)

Additional file 3: References of Included Pilot and Definitive RCTs. (DOCX 30 kb)

## Abbreviations

CI: Confidence intervals
CLEAR NPT: Checklist to Evaluate A Report of Non-Pharmacological Trial
ICC: Intraclass correlation coefficient
n/a: Not applicable
RCT: Randomized controlled trial
