# Supplementary material for: Pilot randomized controlled trials in the orthopaedic surgery literature: a systematic review
Source: BMC Musculoskelet Disord. 2018 Nov 24;19:412. doi: 10.1186/s12891-018-2337-7 (PMC6260657; doi:10.1186/s12891-018-2337-7)
Supplement: Supplementary file 1 — Search Strategy. (DOCX 63 kb) [file 12891_2018_2337_MOESM1_ESM.docx]

**Appendix 1: Search Strategy**

| MEDLINE | EMBASE | PubMed |
| --- | --- | --- |
| 1. exp Pilot Projects/  2. exp Orthopedics/  3. surg$.mp  4. fracture$.mp  5. 2 or 3 or 4  6. 1 and 5  7. limit 6 to (English language and humans and (clinical trial, all or clinical trial, phase i or clinical trial, phase ii or clinical trial, phase iii or clinical trial phase iv or clinical trial or pragmatic clinical trial or randomized control trial))  3076 trials | 1. exp pilot study/  2. exp orthopedics  3. surg$.mp  4. exp fracture  5. 2 or 3 or 4  6. 1 and 5  7. limit 6 to (human and English language and (clinical trial or randomized control trial or phase 1 clinical trial or phase 2 clinical trial or phase 3 clinical trial or phase 4 clinical trial))  2055 trials | 1. Pilot Projects/  2. Orthopedics/  3. surgery  4. fracture  5. 2 or 3 or 4  6. 1 and 5  7. limit 6 to (English language and humans and (clinical trial)  8. limit to Epub ahead of print articles  956 trials |
