# Supplementary material for: Pilot randomized controlled trials in the orthopaedic surgery literature: a systematic review
Source: BMC Musculoskelet Disord. 2018 Nov 24;19:412. doi: 10.1186/s12891-018-2337-7 (PMC6260657; doi:10.1186/s12891-018-2337-7)
Supplement: Supplementary file 2 — Modified Scoring of the CLEAR NPT Scale. (DOCX 87 kb) [file 12891_2018_2337_MOESM2_ESM.docx]

**Appendix 2:** Modified Scoring of the CLEAR NPT Scale.

Asterisk (*) indicates changes from the original scoring method by Somford et al^4^.
