# Supplementary material for: Pilot randomized controlled trials in the orthopaedic surgery literature: a systematic review
Source: BMC Musculoskelet Disord. 2018 Nov 24;19:412. doi: 10.1186/s12891-018-2337-7 (PMC6260657; doi:10.1186/s12891-018-2337-7)
Supplement: Supplementary file 3 — References of Included Pilot and Definitive RCTs. (DOCX 30 kb) [file 12891_2018_2337_MOESM3_ESM.docx]

**Appendix 3:** References of Included Pilot and Definitive RCTs

1. Abbott A, Halvorsen M, Dedering Å. Is there a need for cervical collar usage post anterior cervical decompression and fusion using interbody cages? A randomized controlled pilot trial. Physiother Theory Pract. 2013;29(4):290-300.
2. Adolfsson L, Lindau T, Arner M. Acutrak screw fixation versus cast immobilisation for undisplaced scaphoid waist fractures. J Hand Surg. 2001;26(3):192-5.
3. Bhandari M, Jeray KJ, Petrisor BA, Devereaux PJ, Heels-Ansdell D, Schemitsch EH, et al. A Trial of Wound Irrigation in the Initial Management of Open Fracture Wounds. N Engl J Med. 2015;373(27):2629.
4. Boonen S, Rosen C, Bouillon R, Sommer A, McKay M, Rosen D, et al. Musculoskeletal effects of the recombinant human IGF-I/IGF binding protein-3 complex in osteoporotic patients with proximal femoral fracture: A double-blind, placebo-controlled pilot study. J Clin Endocrinol Metab. 2002;87(4):1593-9.
5. Buse GL, Bhandari M, Sancheti P, Rocha S, Winemaker M, Adili A, et al. Accelerated care versus standard care among patients with hip fracture: the HIP ATTACK pilot trial. CMAJ. 2013;186(1):E52-60.
6. Capa-Grasa A, Rojo-Manaute JM, Rodríguez FC, Martín JV. Ultra minimally invasive sonographically guided carpal tunnel release: An external pilot study. OTSR. 2014;100(3):287-92.
7. Carson JL, Terrin ML, Barton FB, Aaron R, Greenburg, AG, Heck DA, et al. A pilot randomized trial comparing symptomatic vs. hemoglobin‐level‐driven red blood cell transfusions following hip fracture. Transfusion. 1998;38(6):522-9.
8. Chhabra HS, Lima C, Sachdeva S, Mittal A, Nigam V, Chaturvedi D, et al. Autologous mucosal transplant in chronic spinal cord injury: an Indian Pilot Study. Spinal Cord. 2009;47(12):887.
9. Costa ML, MacMillan K, Halliday D, Chester R, Shepstone L, Robinson AH, et al. Randomised controlled trials of immediate weight-bearing mobilisation for rupture of the tendo Achillis. J Bone J Surg Br. 2006;88(1):69-77.
10. Costa ML, Shepstone L, Darrah C, Marshall T, Donell ST. Immediate full-weight-bearing mobilisation for repaired Achilles tendon ruptures: a pilot study. Injury. 2003;34(11):874-6.
11. Darmanis S, Toms A, Durman R, Moore D, Eyres K. A technical innovation for improving identification of the trackers by the LED cameras in navigation-assisted total knee arthroplasty. Comput Aided Surg. 2007;12(4):247-51.
12. De Sèze MP, Bonhomme C, Daviet JC, Burguete E, Machat H, Rousseaux M, et al. Effect of early compensation of distal motor deficiency by the Chignon ankle-foot orthosis on gait in hemiplegic patients: a randomized pilot study. Clin Rehabil. 2011;25(11):989-98.
13. Dutton T, De-Souza R, Parsons N, Costa ML. The timing of tourniquet release and ‘retransfusion’drains in total knee arthroplasty: a stratified randomised pilot investigation. Knee. 2012;19(3):190-2.
14. Ekrol I, Hajducka C, Court-Brown C, McQueen MM. A comparison of RhBMP-7 (OP-1) and autogenous graft for metaphyseal defects after osteotomy of the distal radius. Injury. 2008;39:S73-82.
15. Eskander MBF, Limb D, Stone MH, Furlong AJ, Shardlow D, Stead D, et al. Sequential mechanical and pharmacological thromboprophylaxis in the surgery of hip fractures. Int Orthop.1997;21(4):259-61.
16. Flow Investigators. Fluid lavage of open wounds (FLOW): a multicenter, blinded, factorial pilot trial comparing alternative irrigating solutions and pressures in patients with open fractures. J Trauma Acute Care Surg. 2011;71(3):596-606.
17. Glazebrook M, Younger A, Lalonde, KAA prospective pilot study of B2A-coated ceramic granules (Amplex) compared to autograft for ankle and hindfoot arthrodesis. Foot Ankle Int. 2013;34(8):1055-63.
18. Griffin D, Parsons N, Shaw E, Kulikov Y, Hutchinson C, Thorogood M, et al. Operative versus non-operative treatment for closed, displaced, intra-articular fractures of the calcaneus: randomised controlled trial. BMJ. 2014;349:g4483.
19. Guo Q, Shen Y, Zong Z, Zhao Y, Liu H, Hua X, et al. Percutaneous compression plate versus proximal femoral nail anti-rotation in treating elderly patients with intertrochanteric fractures: a prospective randomized study. J Orthop Sci. 2013;18(6):977-86.
20. Hamdy RC, Montpetit K, Aiona MD, MacKenzie WG, van Bosse HJ, Narayanan U, et al. Safety and efficacy of botulinum toxin A in children undergoing lower limb lengthening and deformity correction: results of a double-blind, multicenter, randomized controlled trial. J Pediatr Orthop. 2016;36(1):48-55.
21. Hamdy R, Montpetit K, Raney E, Aiona M, Fillman R, MacKenzie W, et al. Botulinum toxin type A injection in alleviating postoperative pain and improving quality of life in lower extremity limb lengthening and deformity correction: A pilot study. J Pediatr Orthop. 2009;29(5):427-34.
22. Hey HWD, Hong CC, Long AS, Hee HT. Is hybrid surgery of the cervical spine a good balance between fusion and arthroplasty? Pilot results from a single surgeon series. Eur Spine J. 2013;22(1):116-22.
23. Jordan R, Hao J, Fader R, Gibula D, Mauffrey C. Study protocol: trial of inflation osteoplasty in the management of tibial plateau fractures. EJOST. 2014;24(5):647-53.
24. Kang P, Pei F, Shen B, Zhou Z, Yang J. Are the results of multiple drilling and alendronate for osteonecrosis of the femoral head better than those of multiple drilling? A pilot study. Joint Bone Spine. 2012;79(1):67-72.
25. Kearney RS, Achten J, Parsons NR, Costa ML.The comprehensive cohort model in a pilot trial in orthopaedic trauma. BMC Med Res Methodol. (2011);11(1):1.
26. Kraus VB, Birmingham J, Stabler TV, Feng S, Taylor DC, Moorman CT, et al. Effects of intraarticular IL1-Ra for acute anterior cruciate ligament knee injury: a randomized controlled pilot trial. Osteoarthritis Cartilage. 2012;20(4):271-8.
27. Kuo LC, Yang TH, Hsu YY, Wu PT, Lin CL, Hsu HY, et al. Is progressive early digit mobilization intervention beneficial for patients with external fixation of distal radius fracture? A pilot randomized controlled trial. Clin Rehabil. 2013;27(11):983-93.
28. Lerner T, Bullmann V, Schulte TL, Schneider M, Liljenqvist U. A level-1 pilot study to evaluate of ultraporous β-tricalcium phosphate as a graft extender in the posterior correction of adolescent idiopathic scoliosis. Eur Spine J. 2009;18(2):170-9.
29. Lindsey RW, Wood GW, Sadasivian KK, Stubbs HA, & Block JE. Grafting long bone fractures with demineralized bone matrix putty enriched with bone marrow: pilot findings. Orthopedics. 2006;29(10):939-41.
30. Mahowald ML, Krug HE, Singh JA, Dykstra D. Intra-articular botulinum toxin type A: A new approach to treat arthritis joint pain.Toxicon. 2009;54(5):658-67.
31. Manach YL, Collins G, Bhandari M, Bessissow A, Boddaert J, Khiami F, et al. Outcomes after hip fracture surgery compared with elective total hip replacement. JAMA. 2015;314(11):1159.
32. Mark P, O'Donnell S, Yee G. A pilot clinical evaluation comparing the Mitek bone–tendon–bone cross pin and bioabsorbable screw in anterior cruciate ligament reconstruction fixation, a randomized double blind controlled trial. The Knee. 2008;15(3):168-73.
33. Mauffrey C, McGuinness K, Parsons N, Achten J, Costa ML. A randomised pilot trial of “locking plate” fixation versus intramedullary nailing for extra-articular fractures of the distal tibia. J Bone Joint Surg Br. 2012;1;94(5):704-8.
34. McMorland G, Suter E, Casha S, du Plessis SJ, Hurlbert RJ. Manipulation or microdiskectomy for sciatica? A prospective randomized clinical study. JMPT. 2010;33(8):576-84.
35. Moseley JB, Wray NP, Kuykendall D, Willis K, Landon G. Arthroscopic Treatment of Osteoarthritis of the Knee: A Prospective, Randomized, Placebo-Controlled Trial Results of a Pilot Study. Am J Sports Med. 1996;24(1): 28-34.
36. Nejrup K, de Fine Olivarius N, Jacobsen JL, Siersma V. Randomised controlled trial of extraarticular gold bead implantation for treatment of knee osteoarthritis: a pilot study. Clin Rheumatol. 2008; 27(11), 1363-9.
37. Okcu G, Ozkayin N, Okta C, Topcu I, Aktuglu K. Which implant is better for treating reverse obliquity fractures of the proximal femur: a standard or long nail? Clin Orthop Relat Res. 2013;471(9):2768-75.
38. Pang HN, Yeo SJ, Chong HC, Chin PL, Ong J, Lo NN. Computer-assisted gap balancing technique improves outcome in total knee arthroplasty, compared with conventional measured resection technique. Knee Surg Sports Traumatol Arthrosc. 2011;19(9):1496-503.
39. Paterson K, Nicholls M, Bates D. Intra-articular injection of platelet-rich plasma in patients with knee osteoarthritis: A randomised controlled pilot study. J Sci Med Sport. 2013;16:e90.
40. Ringel F, Stüer C, Reinke A, Preuss A, Behr M, Meyer B, et al. Accuracy of robot-assisted placement of lumbar and sacral pedicle screws: a prospective randomized comparison to conventional freehand screw implantation. Spine. 2012;37(8):E496-501
41. Rojo-Manaute J, Capa-Grasa, A, Chana-Rodríguez F, Perez-Mañanes R, Rodriguez-Maruri G, Sanz-Ruiz P, et al. Ultra-minimally invasive ultrasound-guided carpal tunnel release. J Ultrasound Med. 2016;35(6):1149-57.
42. Sabeti M, Schmidt M, Ziai P, Graf A, Nemecek, E, Schueller-Weidekamm C. The intraoperative use of ultrasound facilitates significantly the arthroscopic debridement of calcific rotator cuff tendinitis. Arch Orthop and Trauma Surg. 2014;134(5):651-6.
43. Sardar Z, Alexander D, Oxner W, Plessis SD, Yee A, Wai EK, et al. Twelve-month results of a multicenter, blinded, pilot study of a novel peptide (B2A) in promoting lumbar spine fusion. J Neurosurg Spine. 2015;22(4):358-66.
44. Shamji MF, Roffey DM, Young DK, Reindl R, Wai EK. A pilot evaluation of the role of bracing in stable thoracolumbar burst fractures without neurological deficit. J Spinal Disord Tech. 2014;27(7):370-5.
45. Storey P, Armstrong D, Dear H, Bradley M, Burke F. Pilot randomised controlled trial comparing C-Trac splints with beta wrist braces for the management of carpal tunnel syndrome. Hand Ther. 2013;18(2):35-41.
46. Vaccaro AR, Patel T, Fischgrund J, Anderson DG, Truumees E, Herkowitz H, et al. A 2-year follow-up pilot study evaluating the safety and efficacy of op-1 putty (rhbmp-7) as an adjunct to iliac crest autograft in posterolateral lumbar fusions. Eur Spine J. 2005;14(7):623-9.
47. Vaccaro AR, Patel T, Fischgrund J, Anderson DG, Truumees E, Herkowitz H, et al. A pilot safety and efficacy study of OP-1 putty (rhBMP-7) as an adjunct to iliac crest autograft in posterolateral lumbar fusions. Eur Spine J. 2003;12(5):495-500.
48. Vaccaro AR, Patel T, Fischgrund J, Anderson DG, Truumees E, Herkowitz HN, et al. A pilot study evaluating the safety and efficacy of OP-1 Putty (rhBMP-7) as a replacement for iliac crest autograft in posterolateral lumbar arthrodesis for degenerative spondylolisthesis. Spine. 2004;29(17):1885-92.
49. Wang Y, Zhou J, Yan F, Li G, Duan X, Pan H, et al. Comparison of arthrodesis with total contact casting for midfoot ulcerations associated with charcot neuroarthropathy. Med. Sci. Monit. 2015;21:2141-8.
50. Wondrasch B, Zak L, Welsch GH, Marlovits S. Effect of accelerated weightbearing after matrix-associated autologous chondrocyte implantation on the femoral condyle on radiographic and clinical outcome after 2 years a prospective, randomized controlled pilot study. Am J Sports Med. 2009;37(1-suppl):88-96.
51. Zehir S, Calbiyik M, Zehir R, Ipek D. Intramedullary repair device against volar plating in the reconstruction of extra-articular and simple articular distal radius fractures; a randomized pilot study. Int J Orthop. 2014;38(8):1655-60.
52. Zehir S, Zehir R, Şahin E, Çalbıyık M. Comparison of novel intramedullary nailing with mini-invasive plating in surgical fixation of displaced midshaft clavicle fractures. Arch Orthop Trauma Surg. 2015;135(3): 339-44.
53. Zhang YZ, Chen B, Lu S, Yang Y, Zhao JM, Liu R, et al. Preliminary application of computer‐assisted patient‐specific acetabular navigational template for total hip arthroplasty in adult single development dysplasia of the hip. Int J Med Robot. 2011;7(4):469-74.
54. Zou J, Zhang W, Zhang CQ. Comparison of minimally invasive percutaneous plate osteosynthesis with open reduction and internal fixation for treatment of extra-articular distal tibia fractures. Injury. 2013;44(8):1102-6.
